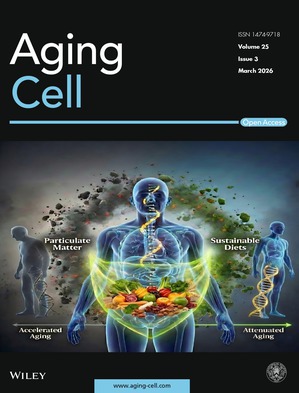# Featured Cover

**DOI:** 10.1111/acel.70450

**Published:** 2026-03-15

**Authors:** Rui Qiang Li, Shou Xin Peng, Rui Hang Zhang, Ting Yu Lu, Wei Sen Zhang, Jiao Wang, Ying Wang, Lin Yang, Shiu Lun Ryan Au Yeung, Tai Hing Lam, Kar Keung Cheng, Lin Xu

## Abstract

Cover legend: The cover image is based on the article *Modifying Role of Sustainable Diets on the Association Between Particulate Matter and Biological Aging: The Guangzhou Biobank Cohort Study* by Rui Qiang Li et al., https://doi.org/10.1111/acel.70422.